# Role of Cefiderocol in Multidrug-Resistant Gram-Negative Central Nervous System Infections: Real Life Experience and State-of-the-Art

**DOI:** 10.3390/antibiotics13050453

**Published:** 2024-05-16

**Authors:** Alessio Sollima, Francesco Rossini, Paola Lanza, Carlo Pallotto, Marianna Meschiari, Ivan Gentile, Roberto Stellini, Angelica Lenzi, Alice Mulé, Francesca Castagna, Silvia Lorenzotti, Silvia Amadasi, Evelyn Van Hauwermeiren, Barbara Saccani, Benedetta Fumarola, Liana Signorini, Francesco Castelli, Alberto Matteelli

**Affiliations:** 1 Department of Clinical and Experimental Sciences, Unit of Infectious and Tropical Diseases, University of Brescia and ASST Spedali Civili di Brescia, 25123 Brescia, Italya.lenzi001@unibs.it (A.L.); a.mule@unibs.it (A.M.); francesco.castelli@unibs.it (F.C.); alberto.matteelli@unibs.it (A.M.); 2 Unit of Infectious and Tropical Diseases, ASST Spedali Civili di Brescia, 25123 Brescia, Italy; p.lanza001@unibs.it (P.L.); silvia.lorenzotti@asst-spedalicivili.it (S.L.); silvia.amadasi@asst-spedalicivili.it (S.A.); efu@hotmail.it (E.V.H.); barbara.saccani@asst-spedalicivili.it (B.S.); benedetta.fumarola@asst-spedalicivili.it (B.F.); liana.signorini@unibs.it (L.S.); 3 Department of Medicine and Surgery, Infectious Diseases Clinic, “Santa Maria della Misericordia” Hospital, University of Perugia, 06132 Perugia, Italy; carlo.pallotto@ospedale.perugia.it; 4 Infectious Diseases Unit, Azienda Ospedaliera-Universitaria of Modena, University of Modena and Reggio Emilia, 41124 Modena, Italy; mariannameschiari1209@gmail.com; 5 Department of Clinical Medicine and Surgery, University of Naples “Federico II”, 80131 Naples, Italy; ivan.gentile@unina.it

**Keywords:** cefiderocol, central nervous system, gram-negative, therapeutic drug monitoring, CNS infections, neurosurgical device infections

## Abstract

Cefiderocol is a new molecule effective against multidrug-resistant (MDR) Gram-negative pathogens. Currently, there is limited evidence regarding the use of cefiderocol in central nervous system (CNS) infections. Data on the cerebrospinal fluid penetration rate of cefiderocol are limited and heterogeneous, and there is no consensus on the dosing scheme of cefiderocol to penetrate the blood–brain barrier. We present a case series and a literature review of CNS infections caused by MDR pathogens that were treated with cefiderocol: some of these patients were treated with different dose schemes of cefiderocol and underwent therapeutic drug monitoring both on plasma and cerebrospinal fluid (CSF). The CSF penetration rates and the clinical outcomes were evaluated.

## 1. Introduction

Cefiderocol is a novel siderophore cephalosporin developed for the treatment of infections caused by multidrug-resistant (MDR) Gram-negative bacteria [1]. The chemical structure of cefiderocol shares some side chains with other cephalosporins: the C-7 side chain is shared with ceftazidime and the C-3 side chain is shared with cefepime. However, its main feature is its chlorocatechol side chain, which chelates ferric iron. Cefiderocol is indeed a next-generation siderophore cephalosporin that exploits two mechanisms of action to enter the bacterial cell: passive diffusion through the outer membrane porins, and active transport through siderophore uptake systems by binding to extracellular iron. Once inside the periplasmic space, iron dissociates, and cefiderocol binds to penicillin-binding proteins, inhibiting the synthesis of bacterial cell wall peptidoglycans, which results in cellular death. This specific mode of action overcomes many bacterial resistance mechanisms. Cefiderocol is active against carbapenem-resistant *Enterobacterales*, *Pseudomonas aeruginosa*, *Acinetobacter baumannii*, *Burkholderia* spp., *Stenotrophomonas maltophilia* and *Elizabethkingia meningoseptica* [2]. It has a half-life of 2−3 h, a protein binding of 58% and renal excretion. As with other beta-lactams, a prolonged infusion is recommended to achieve optimal drug efficacy. The standard intravenous dose is 2 g every 8 h. Renal adjustment is required with an eGFR <60 mL/min. However, patients with critical diseases have hyperdinamic conditions that impact on pharmacokinetics and pharmacodynamics, and this aspect should be taken into account to avert sub-therapeutic drug concentrations [3]. For instance, in glomerular hyperfiltration scenarios (i.e., head trauma, severe burns), a dose escalation to 2 g every 6 h may be recommended [4]. Because of variations in pharmacokinetics/pharmacodynamics, therapeutic drug monitoring is important in optimizing drug efficacy, particularly in critical care contexts. 

Cefiderocol PK/PD is well described and studied in infections such as pneumonia, bacteraemia and urinary tract infections [5,6]. However, the effect of PK/PD variations on central nervous system (CNS) concentrations were not included in registration studies [7,8,9,10]. The literature on this subject is limited and varied [11], and there have only been a few cases featuring CNS infections caused by Gram-negative MDR pathogens treated with cefiderocol reported recently. 

Kufel et al. were the first to describe a case of meningitis caused by carbapenem-resistant *A. baumannii* (CRAB) treated with cefiderocol [12] (Table 1). They also reported cefiderocol concentrations in the CNS with two different treatment regimens: 2 g q6h and 2 g q8h. The CNS penetration rates of both regimens were calculated and estimated as the AUC_CSF_/AUC-free plasma ratio, yielding results of 68% and 60%, respectively. In terms of outcome, the authors report a complete recovery and an absence of side effects.

Luque-Paz et al. reported another case of extensively drug-resistant *P. aeruginosa* ventriculitis treated with cefiderocol [13]. The treatment regimen was 2 g q8h. Drug concentrations measured in plasma and CNS showed a blood–brain barrier penetration of 44%. The patient died of another fungal infection of the CNS.

Marcelo et al. reported a successful case of difficult-to-treat *P. aeruginosa* ventriculitis treated with cefiderocol [14]. They reported a drug penetration in the CNS of 4%, similar to the results obtained by Nau et al. for antibiotic penetration in the CNS without meningeal inflammation (i.e., 2% for penicillins, 10% for cephalosporins and 20% for carbapenems) [15]. In the reported case, despite the low penetration in the CSF, the outcome was successful. However, an increase in the ratio AUC_CSF_/AUC_serum_ was observed in meningeal inflammation (20% in penicillins, 15% in cephalosporins and 30% in carbapenems) [15]. Colombo et al. and Stevenson et al. reported successful outcomes with no data available about cefiderocol concentrations [16,17]. 

To date, no other cases of CNS infection treated with cefiderocol have been described in the literature [18]. In view of the limited amount of data available on the use of cefiderocol in CNS infections, we report five cases from different hospitals regarding CNS infections that were treated with cefiderocol both in a combination regimen and as monotherapy. 

Where available, we also report PK/PD data obtained through therapeutic drug monitoring in both plasma and CSF. Cefiderocol concentrations were quantified through a validated Ultra-High Performance Liquid Chromatography coupled with a Tandem Mass Spectrometry (UHPLC-MS/MS) method with the commercial analytical kit (Kit System Antibiotics^®^, CoQua Lab, Torino, Italy). Briefly, the method was based on plasma protein precipitation, dilution and analysis by UHPLC-MS/MS featuring standardization by a stable-isotope-linked internal standard. Analytical performance was fulfilled according to EMA-ICH guidelines, as previously described [19]. The method was slightly modified by changing the final dilution factor to adapt the sensitivity to the analysis of CSF, with a satisfactory performance in terms of precision and accuracy (CV and bias lower than 15%).

## 2. Case Series

### 2.1. Case 1

**Figure 1:**
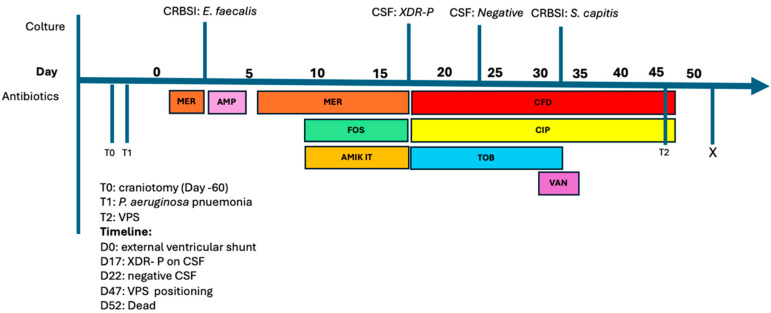
Timeline of nosocomial infections and antimicrobial therapy, Case 1. Abbreviations: CRBSI: catheter-related bloodstream infection; CSF: cerebral spinal fluid; MER: meropenem; AMP: ampicillin; FOS: fosfomycin; AMIK IT: amikacin intrathecal; CFD: cefiderocol; CIP: ciprofloxacin; TOB: tobramycin; VAN: vancomycin; VPS: ventriculoperitoneal shunt; XDR-P: extensively drug-resistant *P. aeruginosa*.

In June 2021, a 60-year-old man was admitted in the neurosurgery unit for a head injury and subarachnoid hemorrhage following an accidental fall at work. He underwent a decompressive craniotomy. The hospital stay was complicated by focal epilepsy and a middle cerebral artery (MCA) aneurysm, which did not necessitate surgical or endovascular intervention. While in the neurosurgical ward, the patient developed nosocomial pneumonia, and *P. aeruginosa* was isolated from the bronchoalveolar lavage. The patient was treated with vancomycin and meropenem with clinical improvement and discharged to a rehabilitation clinic. In August 2021, neurological deterioration was noted, and a head CT scan showed post-hemorrhagic hydrocephalus; the patient was readmitted to the neurosurgical unit on 27 August 2021. An external ventricular shunt was placed. Four days after the procedure, the patient developed a fever and antimicrobial therapy was initiated. No evidence of impairment of liver or kidney function was observed. The patient’s antimicrobial therapy history is summarized in Figure 1, and it includes treatment for intercurrent catheter-related sepsis. Meropenem presumptive therapy was given, initially as a single drug. Later, due to the isolation of Gram-negative bacilli from the CSF, meropenem was given in combination with fosfomycin (16 g/day). When MDR *P. aeruginosa* was identified in the CSF, the antimicrobial therapy was intensified by increasing the fosfomycin dose to 24 g/day and adding intrathecal amikacin. After ten days of treatment, the patient remained pyretic, and treatment was then replaced by cefiderocol (MIC ≤ 2 mcg/mL; 2 g q8h), ciprofloxacin and intrathecal tobramycin. Table 2 shows plasma and cerebrospinal fluid concentrations of cefiderocol on samples collected after 13 days of treatment.

Tobramycin was discontinued after 5 days upon the first CSF culture turning negative, and a ventriculoperitoneal shunt was placed. Cefiderocol and ciprofloxacin were discontinued 3 days later. After 9 days, the patient died of non-infectious causes. 

### 2.2. Case 2

In June 2022, a man who had undergone lung transplantation due to cystic fibrosis was hospitalized for meningitis, ventriculitis, and cerebral/renal abscesses caused by multidrug-resistant *P. aeruginosa*. Despite the clinical presentation, there were no alterations in renal and hepatic function. Treatment with cefiderocol at a dose of 2 g qh6 was initiated, and, after 10 days, significant clinical improvement was observed despite a decrease in CRP levels. Therapeutic drug monitoring was performed, revealing a CSF drug concentration of 7.1 mg/dL (while the plasma concentration at the same time was 62.8 mg/L), resulting in an estimated CSF penetration of 11.3%. MRI imaging revealed complete regression of the brain and renal abscesses. Neurosurgery was performed solely on the largest brain abscess, and the intraoperative cultures revealed the same multidrug-resistant strain of *P. aeruginosa* (MIC to cefiderocol ≤ 2 mcg/mL). The patient was discharged in good health. One year later, the patient was hospitalized for spondylodiscitis at the L1-2 levels and prostatic abscesses. Blood cultures and a bone biopsy showed microbiological isolation of *P. aeruginosa* with the same resistance profile as the previous isolate. Treatment with cefiderocol (2 g qh8) and fosfomycin was given for 3 months, resulting in clinical resolution. No other infectious events occurred after 8 months from the hospital discharge.

### 2.3. Case 3

**Figure 2:**
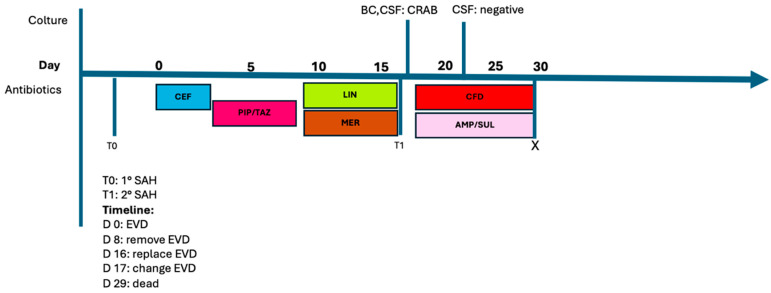
Timeline of nosocomial infections and antimicrobial therapy, Case 3. Abbreviations: AMP/SUL: ampicillin/sulbactam; BC: blood culture; CEF: ceftriaxone; CSF: cerebral spinal fluid; MER: meropenem; EVD: external ventricular device; SAH: subarachnoid hemorrhage; LIN: linezolid; CRAB: carbapenem-resistant *A. baumannii*.

A previously healthy 53-year-old female was admitted to hospital following a subarachnoid hemorrhage (SAH) resulting from the rupture of a posterior-communicating artery aneurysm. Due to the development of hydrocephalus and worsening neurological conditions, an external ventricular drainage (EVD) was inserted. The patient tested negative for colonization by any multidrug-resistant organisms (MDRO) based on nasal and rectal surveillance swabs. Due to the onset of fever, leukocytosis and elevated CRP (no impairment of liver and kidney function), empirical antibiotic therapy was initiated with ceftriaxone, later replaced by piperacillin/tazobactam. On day 8, in the absence of microbiological isolates, treatment was switched to linezolid plus meropenem because of persistent low-grade fever and elevated CRP levels. Although initially showing clinical improvement following EVD removal, on day 16 the patient experienced a second massive SAH, necessitating placement of a new EVD. Concurrently, the patient developed respiratory failure. Carbapenem-resistant *A. baumannii* (sensitive to cefiderocol with MIC ≤ 2 mcg/mL) was isolated from blood cultures (both peripheral and central venous catheter CVC samples), CSF, bronchoalveolar lavage, as well as rectal and nasal swabs. CSF analysis revealed a white blood count of 1720/µL (neutrophils 85%). In light of the in vitro sensitivity of the isolate, high-dose ampicillin/sulbactam plus cefiderocol were initiated and the EVD and CVC were replaced. Therapeutic drug monitoring of cefiderocol was performed and in the following table (Table 3) both plasma and CSF concentrations are reported. After three days of treatment, new blood and CSF cultures turned negative. The patient died on day 29 of hospitalization following another massive SAH. The antimicrobial therapy timeline is showed in Figure 2.

### 2.4. Case 4

**Figure 3:**
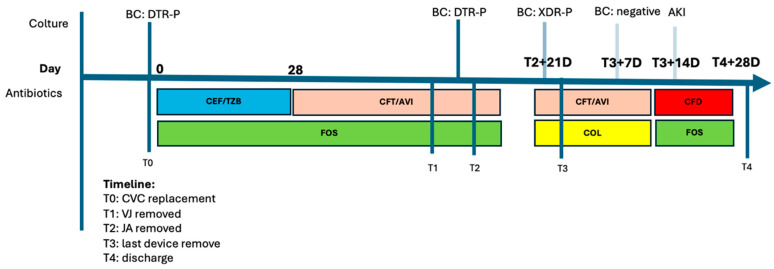
Timeline of nosocomial infections and antimicrobial therapy, Case 4. Abbreviations: BC: blood culture; DTR-P: difficult to treat *P. aeruginosa*; CEF/TZB: ceftolozane/tazobactam; FOS: fosfomycin; CFT/AVI: ceftazidime/avibactam; VJ: ventriculo-jugular; JA: jugulo-atrial; COL: colistin; AKI: acute kidney injury.

A 55-year-old woman with Von Hippel Lindau syndrome and hydrocephalus had a right ventriculoperitoneal shunt positioned in 1994 and a left ventriculo-atrial shunt placed in 2020. In July 2022, she was admitted to hospital with fever and chills. No impairment of liver and kidney function was detected. Blood cultures tested positive for a difficult-to-treat strain of *P. aeruginosa* (sensitive to ceftolozane/tazobactam and ceftazidime/avibactam), and treatment was initiated with ceftolozane/tazobactam 1.5 g qh8 and fosfomycin 8 g qh8 for four weeks. Despite an initial clinical improvement, fever recurred along with persistently positive blood cultures for *P. aeruginosa*. Ceftazidime/avibactam 2.5 g qh8 was given to replace ceftolozane/tazobactam while continuing fosfomycin. As part of the screening procedure, a PET-CT scan was performed, revealing significant contrast uptake of the device. Consequently, the ventriculo-jugular device was removed, while the jugulo-atrial connection was left in place. Despite this intervention, blood cultures remained persistently positive. A brain−neck−chest CT scan was then performed, revealing inflammation in the jugulo-atrial segment that was replaced, leaving a 2 cm portion in place at the left lateral cervical level. Three weeks later, fever recurred, with isolation of an extensively drug-resistant (XDR) strain of *P. aeruginosa* (resistant to ceftolozane/tazobactam and ceftazidime/avibactam, sensitive to cefiderocol with MIC ≤ 2 mcg/mL) in blood cultures. Ceftazidime/avibactam was replaced by colistin, and the remaining device was removed. This treatment led to the resolution of the febrile state and sterilization of blood cultures after one week. Two weeks later, the patient developed acute renal failure, prompting a switch from colistin to cefiderocol while continuing fosfomycin. Complete removal of the device followed by four weeks of combined antibiotic therapy brought a complete clinical, laboratory and microbiological response, and the patient was then discharged home. The antimicrobial therapy timeline is reported in Figure 3.

### 2.5. Case 5

**Figure 4:**
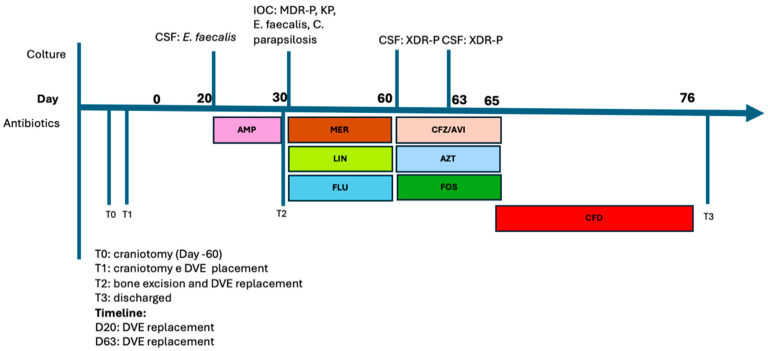
Timeline of nosocomial infections and antimicrobial therapy, Case 5. Abbreviations: CSF: cerebral spinal fluid; AMP: ampicillin; IOC: intraoperative culture; MDR-P: multidrug-resistant *P. aeruginosa*; KP: *K. pneumoniae*; MER: meropenem; LIN: linezolid; FLU: fluconazole; CFZ/AVI: ceftazidime/avibactam; AZT: aztreonam; FOS: fosfomycin; CFD: cefiderocol.

A 74-year-old male with a history of epilepsy underwent a craniotomy for meningioma. Two months later, he underwent a re-intervention for a cerebellar hemorrhagic arteriovenous malformation (AVM). The surgical procedure involved an evacuative craniotomy and the placement of an EVD. Twenty days later, the EVD was replaced due to infection by *E. faecalis* (cultured from CSF and the catheter tip). Ampicillin was initiated. Ten days later, CT scan findings were consistent with osteomyelitis at the surgical site, leading to bone excision and EVD replacement. Cultures obtained from the catheter tip and bone biopsies were positive for MDR *P. aeruginosa* (MIC = 1 for meropenem), ESBL-producing *K. pneumoniae*, *E. faecalis* and *C. parapsilosis* (sensitive to fluconazole). A targeted therapy with high-dose meropenem, linezolid, fluconazole was initiated, resulting in apparent resolution of the surgical site infection. No impairment of liver and kidney function was observed during the course of the treatment. After 30 days, the patient presented with purulent drainage from the EVD (cell count on CSF 304 cells/μL) and worsening neurological symptoms. CSF cultures revealed a XDR strain of *P. aeruginosa* that was resistant to carbapenems. An antimicrobial therapy with ceftazidime/avibactam (2.5 g qh6), fosfomycin and aztreonam was initiated. However, the clinical response was limited. After 3 days, the EVD was replaced, and a new CSF exam showed persistence of *P. aeruginosa* and an increase in CSF cell count. Furthermore, the antibiogram revealed a different resistance profile, with increased MIC to aztreonam (16), ceftazidime/avibactam (8) and fosfomycin (32). The isolate showed susceptibility to cefiderocol (MIC 0.12 mg/L), which was administered on compassionate use. After 7 days, the CSF cultures turned negative. Treatment was continued for 21 days without any adverse events, leading to the normalization of inflammatory markers. The patient was discharged to a rehabilitation clinic. Over the next 60 days, no relapse of infection was observed. The antimicrobial therapy timeline is reported in Figure 4.

## 3. Discussion

Our data suggest that cefiderocol might be a promising candidate for the treatment of CNS infections due to MDR Gram-negative bacteria. We contribute to the literature by providing information in terms of the PK/PD profile of use of the drug in device-associated CNS infections. Our data support the use of an increased dose of cefiderocol (above 2 g q8h) in treatment of CNS infections in light of the high protein-binding ratio of cefiderocol and the mechanisms of hyperfiltration, particularly in the critically ill patient presenting with CNS injuries [3].

Experience with the use of cefiderocol in CNS infections is primarily derived from case reports. Available data are heterogeneous, particularly regarding dose and PK/PD parameters. In our series, the mean penetration in the CSF was estimated from the three cases where therapeutic drug monitoring was conducted and showed a CSF penetration ratio of 8.5% (in line with most of the available literature). The limited data on the penetration rate of cefiderocol into the CNS show significant variability across clinical reports. For instance, Kufel et al. reported much higher CNS penetration rates compared to other authors (68% penetration rate with a 2 g q6h regimen). In contrast, Marcelo et al. report a CNS penetration rate of only 4% [14]. However, the dose does not appear to be a major determinant of the outcome, and additional factors may play a role. For instance, as highlighted by Nau et al., the degree of meningeal inflammation influences drug penetration into the CNS [15,18,20]. Indeed, most of the literature describes cases where CNS infection occurred in the presence of neurosurgical devices, such as ventriculoperitoneal derivations. The dosage of cefiderocol we used, 2 g q6h, is the same dosage used in two out of the three reports that provide this information. Three of our five patients survived. For the two persons who died, the cause of death is not clearly defined in case 1, while patient 3 died from a relapse of subarachnoid hemorrhage. In our series, four of five patients had neurosurgical devices (Table 4). In conclusion, cefiderocol could be a viable therapeutic option for CNS infections caused by multidrug-resistant germs, even in the presence of a device. Effective source control plays a crucial role in the management of assistance-related CNS infections [21]. There is a need to provide clinicians with more robust data through therapeutic drug monitoring, a practice that is not universally available at healthcare centers, which has been shown to lead to improved clinical outcomes [7].

## Figures and Tables

**Table 1:** Summary of case reports on the use of cefiderocol for the treatment of CNS infections. Abbreviations M: Male; F: Female; NA: Not Applicable; CRAB: carbapenem-resistant *Acinetobacter baumanii*; XDR-P: extensively drug-resistant *P. aeruginosa*; DTR-P: difficult to treat *P. aeruginosa*; CPKP: carbapenemase-producing *Klebsiella pneumoniae*; NDM-P: New Delhi metallo beta-lactamase *P. aeruginosa*.

Patient	Age	Sex	Authors, Year of Publication	Pathogen	Neurosurgical Device	Cefiderocol Concentration Data Available	Penetration Ratio	Regime	Outcome
P1	61	F	Kufel et al., 2022	CRAB	Yes	Yes	68% 60%	2 gr q6h 2 gr q8h	Survive
P2	71	NA	Luque-Paz et al., 2022	XDR-P	Yes	Yes	44%	2 gr q8h	Died
P3	63	M	Marcelo et al., 2022	DTR-P	Yes	Yes	4%	2 gr q6h	Survive
P4	44	M	Colombo et al., 2022	CPKP	No	No	NA	2 gr q6h	Survive
P5	41	F	Stevenson et al., 2022	NDM-P	No	Yes/No	NA	1 gr q8h 1.5 gr q8h	Survive

**Table 2:** Therapeutic drug monitoring (TDM) of case report 1. (*) Note that blood contamination in this sample may account for the difference in CSF drug concentration.

	1 h before Infusion	1 h after Infusion
CSF concentration (ng/mL)	3892 (*)	2971
Plasma concentration (ng/mL)	14,172	43,766

**Table 3:** Therapeutic drug monitoring (TDM) of case report 3.

	Plasma (mg/L)	Plasma Free (mg/L)	CSF (mg/L)
2 h before administration	12.45	5.23	0.77
Through	7.13	2.99	0.68
Peak	68.44	28.74	2.18

**Table 4:** Summary of case series. Abbreviations NA: Not Applicable; CRAB: carbapenem resistant *A. baumannii*; XDR-P: extensively drug-resistant *P. aeruginosa*; MDR-P: multidrug-resistant *P. aeruginosa*; CSF: cerebral spinal fluid. (*) cCSF/cPLASMA in P1 was calculated 1 h after infusion; in P3 only the peak concentrations were considered.

Case	Regimen	Pathogen	Neurosurgical Device	Combination Therapy	cCSF/cPLASMA (*)	Outcome
P1	2 g q8 h	XDR-P	Yes	Yes	6.7%	Died
P2	2 g q6 h	MDR-P	No	No	11.3%	Survived
P3	2 g q8 h	CRAB	Yes	Yes	7.5%	Died
P4	2 g q8 h	XDR-P	Yes	Yes	NA	Survived
P5	2 g q6 h	XDR-P	Yes	No	NA	Survived

## Data Availability

No new data were created or analyzed in this study. Data sharing is not applicable to this article due to patient confidentiality.
